# Osteoid osteomas of the hip: a well-recognized entity with a proclivity for misdiagnosis

**DOI:** 10.1007/s00330-023-09765-z

**Published:** 2023-06-07

**Authors:** Doris E. Wenger, Meagan E. Tibbo, Matthew L. Hadley, Rafael J. Sierra, Timothy J. Welch

**Affiliations:** 1 Department of Radiology, Mayo Clinic, 200 First Street SW, Rochester, MN USA; 2 Department of Orthopedic Surgery, Mayo Clinic, 200 First Street SW, Rochester, MN USA

**Keywords:** Osteoid osteoma, Arthralgia, Radiofrequency ablation, Diagnostic errors, Bone neoplasms

## Abstract

**Objectives:**

The diagnosis of osteoid osteomas (OO) about the hip can be challenging as presenting symptoms can mimic other, more common, periarticular pathologies. Our aims were to identify the most common misdiagnoses and treatments, mean delay in diagnosis, characteristic imaging features and provide tips for avoiding diagnostic imaging pitfalls for patients with OO of the hip.

**Methods:**

We identified 33 patients (34 tumors) with OO about the hip who were referred for radiofrequency ablation between 1998 and 2020. Imaging studies reviewed included radiographs (*n* = 29), CT (*n* = 34), and MRI (*n* = 26).

**Results:**

The most common initial diagnoses were femoral neck stress fracture (*n* = 8), femoroacetabular impingement (FAI) (*n* = 7), and malignant tumor or infection (*n* = 4). The mean time from symptom onset to diagnosis of OO was 15 months (range, 0.4–84). The mean time from initial incorrect diagnosis to OO diagnosis was 9 months (range, 0–46).

**Conclusions:**

The diagnosis of OO of the hip is challenging, with up to 70% of cases initially misdiagnosed as a femoral neck stress fracture, FAI, bone tumor, or other joint pathology in our series. Consideration of OO in the differential diagnosis of hip pain in adolescent patients and awareness of the characteristic imaging findings are critical for making an accurate diagnosis.

**Key Points:**

*• The diagnosis of osteoid osteoma of the hip can be challenging, as demonstrated by long delays in time to initial diagnosis and high rates of misdiagnoses which can lead to inappropriate interventions.*

*• Familiarity with the spectrum of imaging features of OO, especially on MRI, is imperative given the increase in the utilization of this modality for the evaluation of young patients with hip pain and FAI.*

*• Consideration of OO in the differential diagnosis of hip pain in adolescent patients and awareness of the characteristic imaging findings, including bone marrow edema and the utility of CT, are critical for making a timely and accurate diagnosis.*

## Introduction

Osteoid osteomas (OO) are benign, bone-forming tumors that most commonly occur in the first to second decades of life [1, 2]. While these tumors can be found throughout the axial and appendicular skeleton, the majority of lesions are found within the metaphyseal and diaphyseal regions of the tibia and femur [3, 4]. Intra-articular occurrences account for approximately 12% of all lesions, with the hip being the most common joint involved [5–8]. Patients classically report night pain that responds readily to non-steroidal anti-inflammatory drugs (NSAIDs) [9]. Additional symptoms include dull achy pain of variable frequency and swelling [2, 10, 11]. For peri-articular lesions in the lower extremities, patients may present with effusions, stiffness, limp, or referred pain to adjacent joints [2, 10, 12–15]. The imaging features of OO on radiographs and CT are widely recognized, typically presenting as a small lytic lesion with or without a tiny central focus of calcification that is surrounded by a variable degree of reactive cortical thickening, chronic benign periosteal new bone formation, and medullary sclerosis [16]. The imaging features of OO on MRI are also well-documented, but may escape detection either due to their small size relative to the larger region of abnormal signal related to the exuberant reactive changes or failure to consider the diagnosis on the differential for a patient with bone marrow edema (BME).

To make an accurate diagnosis of an OO on MRI, it is important to be familiar with the imaging features of BME. BME manifests as a poorly marginated region of T2 (fluid-sensitive) signal hyperintensity that is out of proportion to the findings on T1, where the abnormality could be missed if there is a failure to correlate with the fluid-sensitive images. BME manifests as a hazy grey pattern on T1-weighted MRI with relative preservation of the background of marrow fat signal resulting in signal intensity greater than skeletal muscle. This is in contrast to tumors and infection, which cause confluent replacement of the marrow fat signal on T1 that is typically isointense with or lower in signal intensity than muscle.

Despite their small size and well-documented clinical and imaging features, the diagnosis of osteoid osteomas about the hip can be challenging, with a surprising incidence of delayed or inaccurate diagnoses. In one series of 25 patients with intraarticular lesions, only 2 of the 25 were correctly diagnosed with osteoid osteoma on presentation, resulting in incorrect diagnoses for the other 23 patients lasting anywhere from 4 months to 5 years [17]. Patients presenting symptoms can mimic a variety of other periarticular pathologies including femoroacetabular impingement (FAI), stress fracture, and tumor [14, 16]. The inclusion of OO in the differential diagnosis of hip pain for patients in this age group is an important first step to avoiding misdiagnosis. However, familiarity with the spectrum of imaging features of OO of the hip is also critical to making the correct diagnosis. Given the divergent management of these conditions, as well as their occurrence in young, active patients, accurate and timely diagnosis is crucial for avoiding unnecessary or inappropriate treatment.

Therefore, the aims of our study were to (1) assess the most common misdiagnoses and treatments, (2) define the mean delay in diagnosis, (3) describe the imaging characteristics of periarticular hip OO, and (4) provide tips for avoiding diagnostic imaging pitfalls for patients with OO of the hip.

## Materials and methods

We performed an IRB-approved, retrospective review of 33 patients (34 tumors) diagnosed with osteoid osteomas about the hip. Patients were identified using a prospectively maintained database of a single musculoskeletal radiologist (D.E.W.). Patients were referred to our institution for radiofrequency ablation (RFA) between 1998 and 2020. There were 18 males and 15 females with a mean age of 19 years (range, 7–38 years) at the time of presentation. There were 21 right and 13 left-sided lesions. One patient had two distinct OO, one in each femoral neck, that were diagnosed 2 years apart. Data regarding presenting symptoms, prior diagnoses and treatments, as well as imaging characteristics were manually abstracted from the electronic medical record and were confirmed by two independent reviewers (M.E.T. and M.L.H.).

Thirty of 34 cases were referred after initial evaluation (and in some cases treatment) had been performed elsewhere. Thirty lesions (88%) were in the femoral head/neck, 3 (9%) in the acetabulum, and 1 (3%) in the ischial tuberosity. Imaging studies available for review included radiographs (*n* = 29), CT (*n* = 34), and MRI (*n* = 26). All imaging studies were reviewed by a senior musculoskeletal radiologist (D.E.W.).

### Statistical analysis

Descriptive statistics were utilized to report continuous variables as mean (standard deviation (SD)) or median (range) when appropriate. All analyses were performed using Excel (Microsoft). An alpha level of < 0.05 was deemed to be statistically significant.

## Results

The most common initial diagnoses were femoral neck stress fracture (*n* = 8), FAI (*n* = 7), and malignant tumor or infection (*n* = 4). Additional working diagnoses included Legg-Calve-Perthes, patellar tendinitis, post-traumatic pain, sacroiliac joint dysfunction, and inflammatory arthritis. Seven patients had no prior diagnosis and three were correctly diagnosed as having OO (Table 1). Prior non-operative treatments included a trial of non-weight bearing on crutches (*n* = 5), intra-articular hip injections (*n* = 2), epidural spinal injection (*n* = 1), and activity modification with physical therapy (*n* = 5). Prior operative interventions included open reduction internal fixation for a femoral neck stress fracture (*n* = 2), hip arthroscopy with femoral neck osteochondroplasty followed by reverse periacetabular osteotomy (PAO) (*n* = 1), arthroscopic femoral neck and acetabular osteochondroplasty (*n* = 1), arthroscopic labral repair (*n* = 1), arthroscopic synovial biopsy and synovectomy for presumed inflammatory arthropathy (*n* = 1), and attempted RFA (*n* = 1) (Table 2).

**Table 1:** Initial diagnoses

Diagnosis	***n***
Femoral neck stress fracture	8
Femoral acetabular impingement	7
No indicated prior diagnosis	7
Infection/non-OO oncologic process	4
Osteoid Osteoma	3
Legg-Calve-Perthes disease	1
Patellar tendinitis	1
Post-traumatic pain	1
Sacroiliac joint dysfunction	1
Inflammatory arthritis	1

**Table 2:** Operative interventions prior to OO diagnosis and RFA

Operative intervention	*n*
Open reduction internal fixation	2
Reverse periacetabular osteotomy	1
Radiofrequency ablation	1
Arthroscopic acetabular and femoral neck osteochondroplasty	1
Arthroscopic labral repair	1
Arthroscopic synovial biopsy and synovectomy	1

The mean time from symptom onset to diagnosis of OO was 15 months (range, 0.4–84 months). The mean time from initial incorrect diagnosis to OO diagnosis was 9 months (range, 0–46 months). No data are available regarding the number of visits or subspecialty of the treating physician prior to patients’ referral to our institution. Twenty-six of 34 (76%) initial consultation notes at our institution indicated a history of “night pain” about the hip, with pain relief associated with the administration of an anti-inflammatory medication documented in 26/34 (76%) of these notes. Symptoms resolved in all but one patient following RFA utilizing a Cool-tip probe (Covidien). Core biopsies were obtained in all patients at the time of RFA with an Osteo-site 11–14G bone biopsy needle set (Cook Medical, Inc.) yielding a diagnosis of OO in 17 cases (52%).

All but one MRI exam showed BME in the head/neck of the femur (*n* = 21), acetabulum (*n* = 3), and ischial tuberosity (*n* = 1). The osteoid osteoma was not identified prospectively and the BME was attributed to other pathology for 21 of 26 (81%) MRI exams. The OO was evident on all CT exams and in retrospect on 12 of 29 radiographs and 23 of 26 MRI studies.

**Fig. 1:**
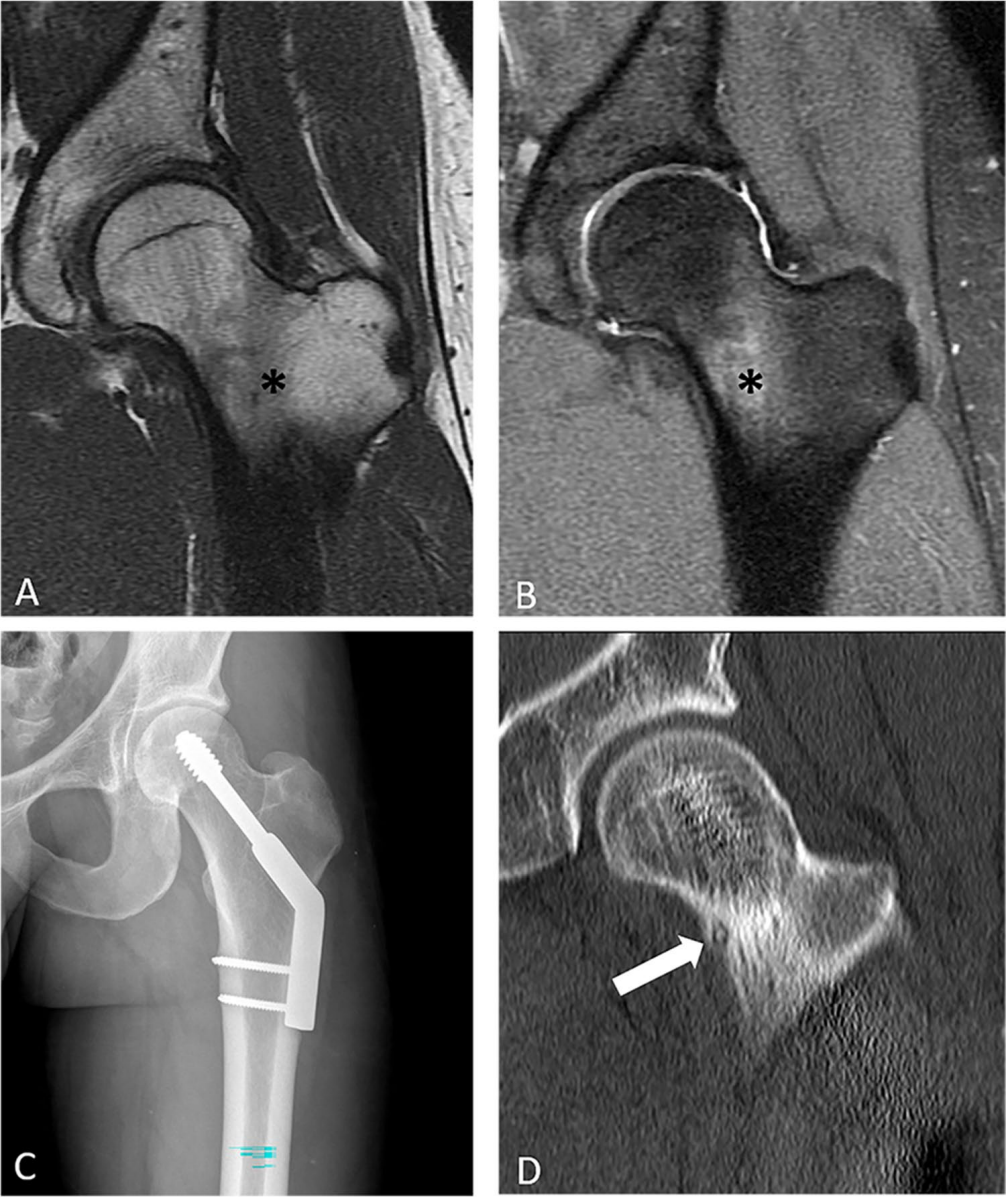
Coronal T1 (**A**) and STIR (**B**) MR images of the left hip of a 26-year-old male show bone marrow edema in the femoral neck (asterisk). Patient was diagnosed with a stress fracture and treated with dynamic hip screw fixation (**C**). Symptoms were unchanged postoperatively. Coronal CT obtained to evaluate for a source of pain and hardware complications (**D**) shows a tiny intracortical OO in the femoral neck anteriorly (arrow) that was successfully treated with RF ablation

**Fig. 2:**
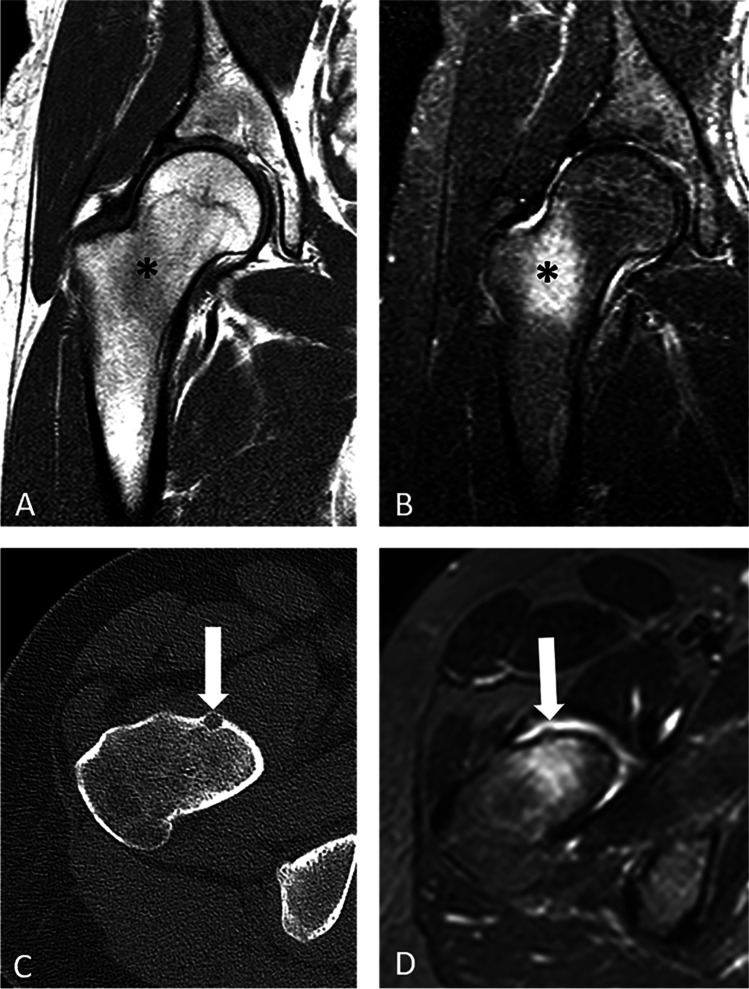
A 19-year-old female with OO of the right femoral neck. Radiographs of the right hip were negative (not shown). Coronal T1 (**A**) and fat-suppressed T2-weighted (**B**) MR images show bone marrow edema in the femoral neck (asterisk). She was initially diagnosed with a femoral neck stress fracture, then with FAI, and was sent for metabolic bone disease evaluation prior to being diagnosed with a 6 mm OO that was confirmed on axial CT (**C**), In retrospect, the OO was evident on the axial T2 weighted MRI (**D**), but easy to miss if not entertaining the diagnosis (arrow)

**Fig. 3:**
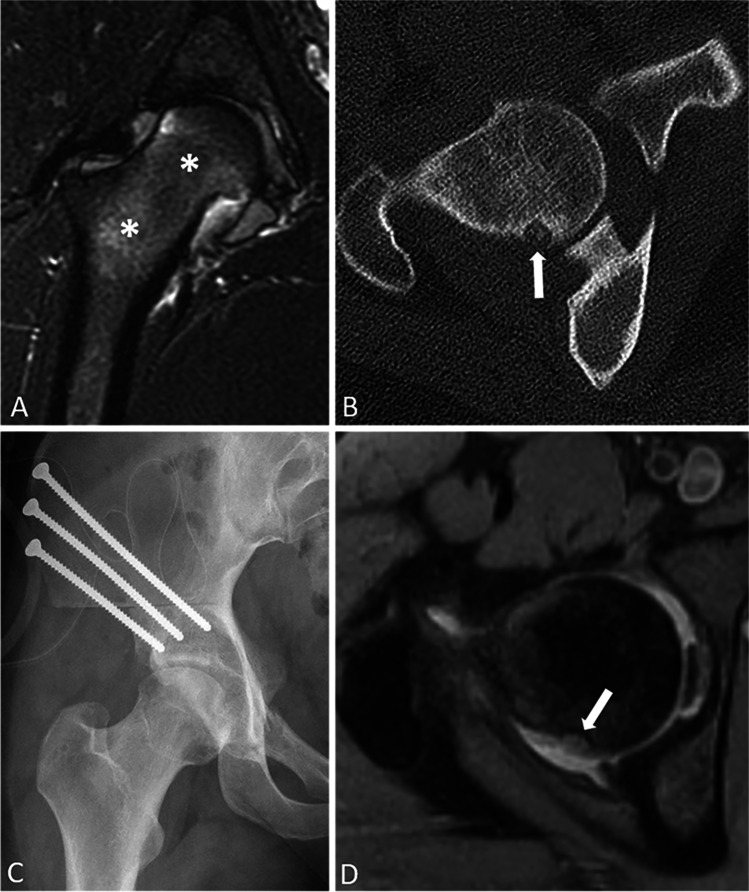
Coronal STIR MR image of the right hip (**A**) of an 18-year-old female shows a joint effusion, synovitis, and bone marrow edema in the head and neck of the femur (asterisks), along with a labral tear and cam deformity (not shown). Patient was treated with hip arthroscopy and head-neck osteoplasty with no appreciable change in pain. Axial CT (**B**) obtained for pre-operative planning for subsequent periacetabular osteotomy (**C**) shows an OO near the head-neck junction of the femur posteriorly (arrow). In retrospect, the OO was visible on the original axial proton density MRI (**D**), but easy to miss if not entertaining the diagnosis on the differential. Patient reported pain at night relieved with NSAIDs, which was only relieved following RF ablation of the OO

**Fig. 4:**
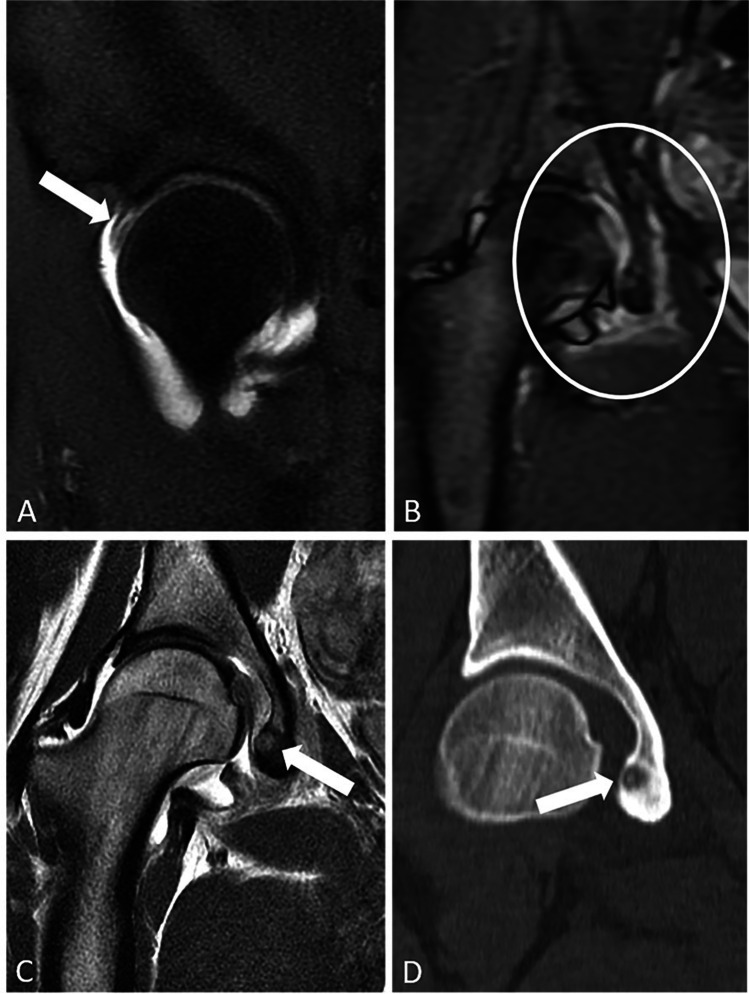
A 16-year-old female with a clinical diagnosis of FAI was referred for an MRI arthrogram to evaluate for a labral tear. Sagittal T1 weighted MRI (**A**) with intra-articular gadolinium shows an anterosuperior labral tear (arrow). Large field-of-view coronal STIR image of pelvis and hips (**B**) shows asymmetric increased T2 signal compatible with edema in the bone and soft tissues along the medial wall of the right acetabulum (circle), which would be unexpected with a diagnosis of FAI. Coronal PD image of the hip shows a tiny round lesion in the medial wall of the acetabulum near the epicenter of the edema (**C**), which was seen to better advantage and confirmed to represent an OO with marked surrounding sclerosis on coronal CT (**D**)

In our series, BME was incorrectly attributed to a stress fracture in 8 cases, despite the lack of an identifiable fracture on the images in all cases (Fig. 1) and despite failure to question the initial diagnosis of stress fracture when there was no change in BME or change in symptoms in response to conservative treatment that included rest and/or immobilization (Fig. 2). There were 7 cases in our series of OO that were diagnosed with FAI, 3 of whom had the previously described surgical procedures. Whether the pain was caused by the OO and/or the FAI can be difficult to determine in retrospect, but at least one of the patients who had two prior procedures for FAI, including a PAO, had night pain prior to the surgeries that was only relieved following subsequent RFA of the OO (Fig. 3). In this study, one of the factors that was helpful to avoid missing the OO in a patient otherwise referred with a working diagnosis of FAI was to recognize BME in an unexpected anatomic location for FAI. In our series, the BME was located in the medial aspect of the femoral neck, medial wall of the acetabulum (Fig. 4), ischial tuberosity, and in one, a large region of the supra-acetabular ilium, all of which would be atypical locations for BME in patients with FAI.

There were 22 patients (65%) in our series with no evidence of a hip joint effusion, including 17 patients with an intra-articular OO. Twelve patients (35%) with intra-articular OO had hip joint effusions, 6 with small (17%), 5 with moderate (15%), and one with large (3%) joint effusions. The MRI of the patient with the large joint effusion was interpreted as being suspicious for inflammatory arthritis and the OO was only identified in retrospect.

Of the 26 MRI exams, 3 were performed with intravenous gadolinium, 2 with intra-articular gadolinium, and 21 (81%) without IV or intra-articular gadolinium. All of the CT exams were performed without IV contrast. Of the 3 MRI exams performed with intravenous gadolinium, none of the OO were identified prospectively.

**Fig. 5:**
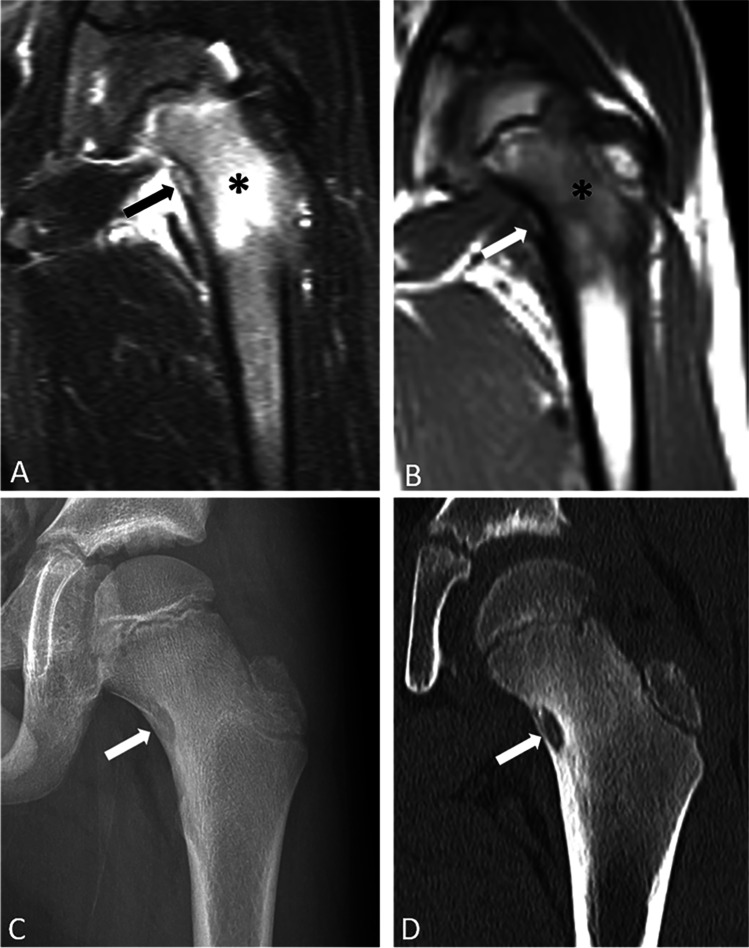
A 10-year-old male was referred with a working diagnosis of osteosarcoma. Coronal T1 (**A**) and STIR (**B**) MR images show a large joint effusion and increased T2 signal in the neck and intertrochanteric region of the proximal femur that is out of proportion to the findings on T1 where there is relative preservation of marrow fat signal (asterisk). Recognizing a bone marrow edema pattern, closer inspection of the MRI images reveals an obvious round to ovoid lesion in the femoral neck medially (arrow), that was missed given the lack of consideration of the diagnosis on the differential. Subsequent AP radiograph (**C**) and coronal CT (**D**) confirm the diagnosis of an intracortical OO (arrow)

There were 4 cases in our series that were misdiagnosed as tumor or infection, including one patient who was referred with a working diagnosis of osteosarcoma, related to failure to recognize the BME pattern and consider OO on the differential diagnosis (Fig. 5). This case also illustrates the value of radiographs, since they showed an obvious lytic lesion in the medial femoral neck and were only obtained after the MRI when the patient was referred with a presumptive cancer diagnosis.

## Discussion

Despite their small size and well-documented clinical and imaging features, the diagnosis of osteoid osteoma in the hip can be challenging. In our series, up to 70% of cases were initially misdiagnosed as a femoral neck stress fracture, FAI, bone tumor, or other joint pathology. This led to lengthy delays in diagnosis and potentially avoidable operative intervention in 18% of cases. Data in the present study suggests that consideration of OO in the differential diagnosis of hip pain, especially in adolescent and young adult patients, may lead to a reduction in incorrect diagnoses and more rapid, appropriate therapeutic intervention. Familiarity with the spectrum of imaging features of OO, especially on MRI, is imperative given the increase in the utilization of this modality for the evaluation of young patients with hip pain and FAI.

The increased propensity for misdiagnosis of OO about the hip is likely multifactorial in nature. Similarities in clinical characteristics between OO and other hip joint pathologies, as well as the overlap of age groups, could represent significant contributors to the misdiagnosed cases identified in our series and others. Compared to extraarticular lesions, intraarticular occurrences of OO are reported to be more likely to be associated with joint stiffness, effusions, synovitis, and even contractures [17]. In our series, the presence of a hip joint effusion and/or synovitis was not helpful in suggesting the diagnosis, as the majority of patients (65%) showed no evidence of a hip joint effusion, including 17 patients with an intra-articular OO. Twelve patients (35%) with intra-articular OO had hip joint effusions, 6 with small (17%), 5 with moderate (15%), and only one with large (3%) joint effusions. The MRI of the patient with the large joint effusion was interpreted as being suspicious for inflammatory arthritis and the OO was only identified in retrospect. Our results did not concur with a previous study by Germann et al. that reported a perfect sensitivity and specificity (100%) and a high negative predictive value of 85% for joint effusion and synovitis in 21 patients with intra-articular OO [18].

Pain, often greater at night, is the classic presenting symptom of both intraarticular and extraarticular OO lesions. However, this pain pattern, along with symptoms such as decreased ROM and a limp, can often overlap with other common hip pathologies including FAI and stress fracture, and may even raise concern for inflammatory arthritides [2, 10, 12–14]. In our study, 76% of initial consultation notes at our institution indicated that the patient had a history of “night pain” about the hip, with that same percentage reporting pain relief associated with anti-inflammatory medication administration, most often a NSAID. May et al., in a series looking specifically at a cohort of children and adolescents with OO about the hip, reported “night pain” and “symptom relief with NSAIDs” on presentation in 90% and 88% of patients respectively [1]. These data correlate with our cohort and indicate that an important step in avoiding misdiagnosis of OO is to entertain the diagnosis itself, especially in a young patient with hip pain that is worse at night and responds to NSAIDs.

Conventional radiographs are generally considered to be the initial imaging modality of choice for young patients presenting with hip pain. Although they may identify the aforementioned typical radiographic features of an OO, they may also be radiographically occult. This is especially true for lesions that are intra-articular, as the relatively thin surrounding periosteum results in a less robust osteoblastic response, and decrease in visible sclerosis with radiography [1, 12]. The limitations of this imaging modality in making an accurate diagnosis of OO were echoed in our data, as only 41% of OO lesions were retrospectively identified on radiographs.

Advanced imaging in patients with undifferentiated hip pain often begins with an MRI, which provides a comprehensive evaluation of the bones and soft tissues, with the added benefit of avoiding radiation exposure. However, MRI in the setting of OO about the hip can also demonstrate nonspecific findings, including joint effusion, synovitis, labral tears, and bone marrow edema, which may lead to erroneous diagnoses. In our series, all but one MRI exam demonstrated bone marrow edema, which was attributed to other pathology in 81% of cases. The osteoid osteoma was missed on the original MRI for 81% of cases. During re-review, OO was visible on MRI in 23/26 (88%) of cases. Increased risk for misdiagnosis has been reported in other series when MRI is used as the primary imaging modality. In a review of 43 patients by Davies et al., they reported that the potential for a missed diagnosis of OO was 35% based solely on the MRI exam [19]. Although dynamic contrast-enhanced MRI has been reported to improve the sensitivity for detection of OO by increasing their conspicuity by Liu et al., the majority of the MRI exams in our cohort were performed without intravenous or intra-articular gadolinium (81%) [20]. Of the 3 MRI exams performed with intravenous gadolinium, none of the OO were identified prospectively. Given that the diagnosis of OO was missed prospectively in 82% and identified in retrospect for 88% of cases, our results indicate that the diagnosis of OO can be made even with an unenhanced MRI when considering the diagnosis.

Hosalker et al., in a series assessing the diagnostic accuracy of CT versus MRI for OO in children, found that only 3% of lesions were confirmed to be OO with MRI, as compared to 67% with CT scan [21]. When considering the diagnosis in the differential, CT is the most accurate and sensitive imaging modality for making the diagnosis of an OO [22–24]. Our data demonstrated the superiority of CT over MRI in detecting OO, with all lesions (34/34) being clearly visible on all CT exams. Although MRI is a commonly utilized and comprehensive imaging modality in the evaluation of young patients with hip pain and in whom radiation exposure is understandably avoided when possible, our data highlight the diagnostic value of early CT imaging in the appropriate clinical setting to facilitate an accurate diagnosis.

Given the increase in the utilization of MRI to evaluate patients with hip pain (especially FAI) in a similar age group and anatomic location for patients presenting with OO, familiarity with their MRI imaging features is paramount. Specifically, the ability to recognize the BME pattern on MRI is critical for distinguishing between OO and lesions that cause confluent replacement of marrow fat on T1 such as tumor and infection. It is also important to note the anatomic location of the BME since the edema associated with many of the OO in our series would be atypical in the setting of FAI. Therefore, when confronted with significant BME without an identifiable stress fracture, a meticulous search for a small round lesion near the epicenter of the edema should ensue. If an OO is not detected and the clinical history and edema pattern suggest the diagnosis, a CT is the imaging modality of choice to confirm the diagnosis of OO. Given the importance of imaging, as it relates to making an accurate diagnosis of OO about the hip, radiologists may be the first to suggest the diagnosis.

The diagnostic challenge presented by OO about the hip can also lead to long delays in making an accurate diagnosis. In our series, the mean delay from the time of symptom onset to diagnosis of OO was 15 months, with a maximum delay of 84 months (7 years). The interval of time between the patient receiving their first incorrect diagnosis and the correct diagnosis of OO was 9 months, with a maximum delay of 46 months (~ 4 years). Delays in establishing the correct diagnosis for this condition have also been reported in the literature. Szendroi et al. cited a mean delay from symptom onset to diagnosis of 26.6 months for intra-articular OO lesions, and a delay of 8.5 months for extra-articular lesions [25]. In a series of fifty children and adolescents with OO about the hip, May et al. found a mean delay in diagnosis of greater than 6 months in 43% of their patients [1]. They identified female sex and a lack of limping as findings significantly associated with a delay. While we do not know how many providers the patients in our series saw prior to being evaluated at our institution, we do know that they did see an orthopedic surgeon in consultation if they had some form of operative intervention, which occurred in 6 of the 34 cases. This delay may be mitigated to some extent by selecting appropriate advanced imaging, based on the patients’ radiographic and clinical findings.

Limitations of this study are primarily related to its small sample size, as well as limitations inherent in a retrospective review. Additionally, we do not have data regarding the number of visits or subspecialty of the treating physician prior to patients’ referral to our institution. This may have provided additional insight into the rationale behind a patients’ prior workup.

## Conclusion

In conclusion, consideration of OO in the differential diagnosis of hip pain in adolescent patients as well as awareness of the characteristic imaging findings, including BME and the utility of CT, are critical for making a timely and accurate diagnosis.

## Abbreviations

BME: Bone marrow edema
FAI: Femoroacetabular impingement
NSAID: Non-steroidal anti-inflammatory drug
OO: Osteoid osteoma
PAO: Periacetabular osteotomy
RFA: Radiofrequency ablation
SD: Standard deviation

## Declarations

### Guarantor

The scientific guarantor of this publication is Dr. Doris Wenger.

### Statistics and biometry

No complex statistical methods were necessary for this paper.

### Methodology

- retrospective
- observational
- one institution
